# An unexpected case of recurrence of pulmonary embolism in a patient recovered from COVID19 in full regimen dose of direct oral anticoagulant drug

**DOI:** 10.1186/s12890-021-01453-2

**Published:** 2021-03-24

**Authors:** Emilia D’Elia, Mauro Gori, Aurelia Grosu, Annamaria Iorio, Ferdinando Luca Lorini, Anna Falanga, Fabiano Di Marco, Michele Senni

**Affiliations:** 1 Cardiovascular Department, Hospital Papa Giovanni XXIII, Piazza OMS 1, Bergamo, Italy; 2 Department of Medicine and Surgery, University of Milan, Milan, Italy; 3 Department of Health Sciences, University of Milan, Milan, Italy

**Keywords:** COVID-19, Pulmonary Embolism, Complications, Direct Oral Anticoagulation, Case Report

## Abstract

**Background:**

Coronavirus Disease 2019 (COVID-19) is a pandemic affecting all countries in the world. Italy has been particularly afflicted by the health emergency, and since the peak phase has passed, major concern regarding medium to long term complications due to COVID-19 is arising. Little is known in literature regarding thromboembolic complications once healed after COVID-19.

**Case presentation:**

A 51-year-old patient recovered from COVID-19 pneumonia complicated by pulmonary embolism (PE) came to the hospital for palpitations and chest pain. Although he was on treatment dose of direct oral anticoagulation (DOAC), massive recurrent PE was diagnosed.

**Conclusion:**

In the early post COVID-19 era, the question remains regarding the efficacy of DOACs in COVID-19 patients.

## Background

Severe acute respiratory syndrome coronavirus 2 (SARS- CoV-2) caused by COVID-19 is a pandemic affecting all countries in the world [1]. Bergamo, Italy, has been particularly afflicted by this pandemic, earning the sad record of the city with the highest mortality in the world. Fortunately, the peak phase has passed, and we are almost back to normality. If on the one hand it is reassuring that new cases are decreasing, and the patients recovered are clearly increasing, on the other hand it is worrisome to assist to unexpected medium-term complications.

## Case presentation

On April 29, 2020, a 51-year-old man presented to the Emergency Room (ER) with palpitations and chest pain. He was in a rehabilitation center after being discharged five days earlier from the hospital, where he had been hospitalized for COVID-19 pneumonia, complicated by right PE. He was initially treated with unfractionated heparin (UFH) and warfarin, and, once improved and hemodynamically stable, warfarin was replaced with dabigatran 150 mg twice a day. After three consecutive negative COVID-19 swabs, the patient was discharged on curative regimen of DOAC. In addition to dabigatran, his therapy included betablockers, corticosteroids, levothyroxine, benzodiazepines.

Examination findings at that ER admission were as follows: blood pressure (BP) 90/60 mmHg, heart rate 97 beats/min, respiratory rate 20 breaths/min, temperature 37.1 °C, normal oxygen saturation (SaO2) on room air. On physical examination, he had an irregularly irregular heart rate, normally transmitted vesicular murmur, no findings suggestive for heart failure (HF). The electrocardiogram (ECG) showed atrial fibrillation, and intravenous amiodarone bolus plus infusion was started. Suddenly, he started to complain shortness of breath. He was found to be in respiratory distress, with hypotension (BP 85/55 mmHg) and SaO2 85%. Oxygen 4 l/min was placed and, due to persistent desaturation, non-rebreather oxygen mask (15 l/min) was started.

**Fig. 1:**
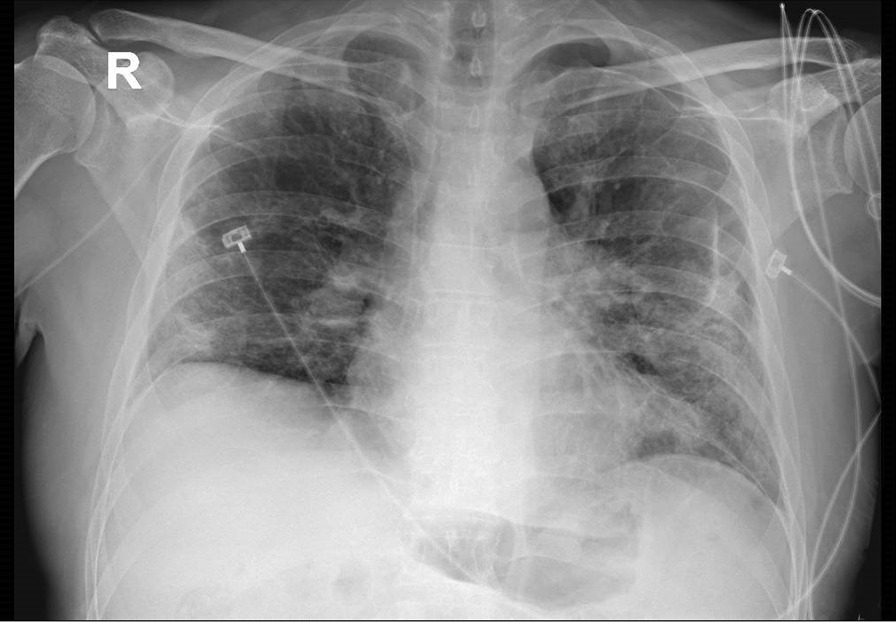
Chest X Ray showing bilateral interstitial infiltrates with accentuation of the vascular plot, in the absence of pleural effusion

Laboratory investigation revealed an increase of high-sensitive troponin (90 ng/L, normal range < 54 ng/L), an elevated D-Dimer (3499 ng/ml), an increase of the white blood cell count (26,000), and a normal C-reactive protein (0.2 mg/L). Chest x-ray showed bilateral interstitial infiltrates with accentuation of the vascular plot, in the absence of pleural effusion (Fig. 1). Arterial blood gas at the time of the onset of dyspnea showed PH 7.38, PaCO_2_ 22 mmHg, PaO_2_ 48 mmHg, and bicarbonate 20 mmol/L. A reservoir 15 l/min was placed. On 70% FiO_2_, arterial blood gas showed PH 7.44, PaCO_2_ 37.8 mmHg, PaO_2_ 151 mmHg, and bicarbonate 16 mmol/L. An ECG showed a tachycardia, with a new onset right bundle branch block, S1, and Q3. At the transthoracic echocardiography, right-sided chambers were severely dilated. Due to the sudden clinical deterioration, thrombolysis with tissue plasminogen activator (tPA) was immediately performed in the suspicion of recurrent PE. The patient had a rapid beneficial effect with restoration of stable hemodynamic parameters.

**Fig. 2:**
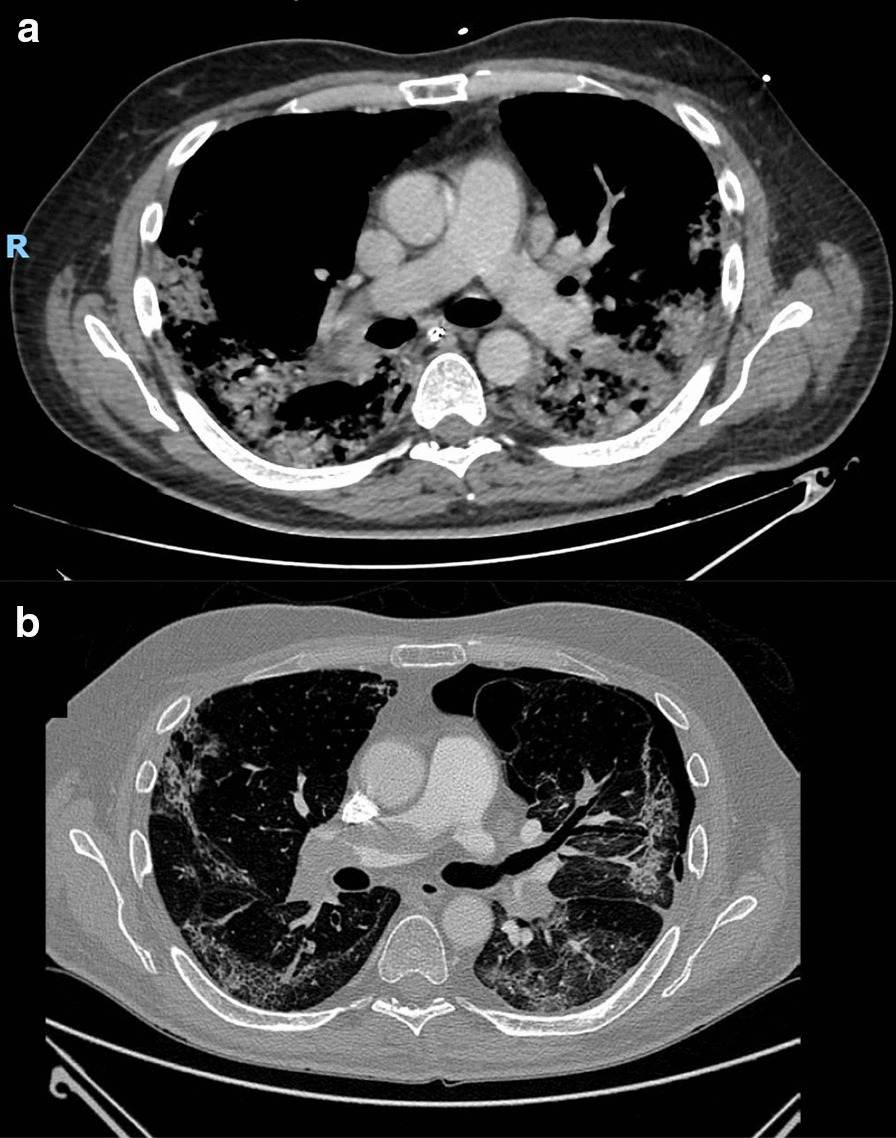
Computed tomography angiogram during the first hospital admission showing a focal filling defect affecting the pulmonary artery branch for the lower right lobe with thromboembolic outbreak (**a**). Computed tomography angiogram at the time of hospital re-admission showing extensive defect of endoluminal filling of the right and left pulmonary artery with extension to their main branches (**b**)

After infusion of tPA, a computed tomography angiogram was performed, which documented marked defect of endoluminal filling of the right and left pulmonary artery with extension to their main branches referable to acute PE, more extensive than the previous one (Fig. 2). The patient was than admitted to the Intensive Care Unit and UFH was started intravenously. During the in-hospital stay, the patient remained hemodynamically stable, in sinus rhythm, and apyretic. His COVID19 swab test resulted negative. To exclude venous thromboembolism (VTE), a venous echo-Doppler ultrasound was performed, which resulted negative. After collegial discussion, it was decided to continue for one month with LMWH and then, if there will be more clearer data on the matter, to start warfarin.

## Discussion

Reports of acute PE associated with COVID-19 have emerged more and more in the literature [2–5], and frequency of PE in the COVID-19 era seems to be extremely higher than that in the same control period of 2019 [6]. Nearly 20% of COVID-19 patients present with severe coagulation abnormalities, which may occur in almost all of the critically ill COVID-19 cases, and can negatively affect prognosis. It has also been reported that markedly elevated D-dimer, fibrinogen degradation products and prothrombin were very common in COVID-19-related deaths [7, 8]. Mini autopsies have shown microthrombi in lungs, in liver, and in the entire vascular system, suggesting that severe endothelial dysfunction, driven by the cytokine storm and induced by hypoxemia, leads to disseminated intravascular coagulation, causing thromboembolic complications [9]. However, in the early the post-COVID-19 era, there are no data regarding the likelihood of recurrent PE in healed COVID-19 patients, already on treatment dose anticoagulant therapy. Nowadays, in all severe COVID-19 patients and in those suspected for VTE, if there is no anticoagulation contraindication, it is suggested to start curative anticoagulant therapy. The first-line treatment is based on parenteral anticoagulation with low molecular weight heparin (LMWH), or UFH by venous infusion in case of severe renal impairment, with a regular monitoring for anticoagulation dose adjustment. The DOACs are an option only after the acute phase if the patient is more stable with no more treatment interfering with CYP3A4, and no major comorbidities [10].

As described in our case report, nonetheless, recurrence of PE can occur in the early post COVID-19 era despite curative regimen of DOACs. It is still not yet clear how to behave with the anticoagulation regimen to prevent these mid-term complications. Specifically, it remains to be clarified whether a strategy with LMWH embraced to warfarin is superior to the treatment dose of DOACs. Further randomized studies are needed to assess the best prophylaxis and treatment of this worrying condition [11, 12].

In conclusion, in the early post COIVD-19 discharge, it is of fundamental importance to recognize the medium-term risk of thromboembolic complications, especially in the most critical patients. To this regard, it would be advisable to perform randomized clinical trials to demonstrate the best anticoagulant regimen to be dedicated to these patients.

## Data Availability

Data of the patient available from the corresponding author.

## Abbreviations

COVID-19: Coronavirus Disease 2019
PE: Pulmonary embolism
DOAC: Direct oral anticoagulation
Sars-CoV-2: Severe acute respiratory syndrome coronavirus 2
ER: Emergency room
UFH: Unfractionated heparin
BP: Blood pressure
SaO2: Oxygen saturation
HF: Heart failure
ECG: Electrocardiogram
tPA: Tissue plasminogen activator
VTE: Venous thromboembolism
LMWH: Low molecular weight heparin
